# Proteomic landscape of Alzheimer’s Disease: novel insights into pathogenesis and biomarker discovery

**DOI:** 10.1186/s13024-021-00474-z

**Published:** 2021-08-12

**Authors:** Bing Bai, David Vanderwall, Yuxin Li, Xusheng Wang, Suresh Poudel, Hong Wang, Kaushik Kumar Dey, Ping-Chung Chen, Ka Yang, Junmin Peng

**Affiliations:** 1 Departments of Structural Biology and Developmental Neurobiology, St. Jude Children’s Research Hospital, 38105 Memphis, TN USA; 2 Center for Proteomics and Metabolomics, St. Jude Children’s Research Hospital, 38105 Memphis, TN USA; 3 Current address: Center for Precision Medicine, Department of Laboratory Medicine, Nanjing Drum Tower Hospital, The Affiliated Hospital of Nanjing University Medical School, Jiangsu 210008 Nanjing, China; 4 Current address: Department of Biology, University of North Dakota, ND 58202 Grand Forks, USA

**Keywords:** Alzheimer’s disease, Pathogenesis, Biomarker, Proteomics, Proteome, PTM, Mass spectrometry, Tau, Abeta, Amyloidome

## Abstract

Mass spectrometry-based proteomics empowers deep profiling of proteome and protein posttranslational modifications (PTMs) in Alzheimer’s disease (AD). Here we review the advances and limitations in historic and recent AD proteomic research. Complementary to genetic mapping, proteomic studies not only validate canonical amyloid and tau pathways, but also uncover novel components in broad protein networks, such as RNA splicing, development, immunity, membrane transport, lipid metabolism, synaptic function, and mitochondrial activity. Meta-analysis of seven deep datasets reveals 2,698 differentially expressed (DE) proteins in the landscape of AD brain proteome (*n* = 12,017 proteins/genes), covering 35 reported AD genes and risk loci. The DE proteins contain cellular markers enriched in neurons, microglia, astrocytes, oligodendrocytes, and epithelial cells, supporting the involvement of diverse cell types in AD pathology. We discuss the hypothesized protective or detrimental roles of selected DE proteins, emphasizing top proteins in “amyloidome” (all biomolecules in amyloid plaques) and disease progression. Comprehensive PTM analysis represents another layer of molecular events in AD. In particular, tau PTMs are correlated with disease stages and indicate the heterogeneity of individual AD patients. Moreover, the unprecedented proteomic coverage of biofluids, such as cerebrospinal fluid and serum, procures novel putative AD biomarkers through meta-analysis. Thus, proteomics-driven systems biology presents a new frontier to link genotype, proteotype, and phenotype, accelerating the development of improved AD models and treatment strategies.

**Supplementary Information:**

The online version contains supplementary material available at 10.1186/s13024-021-00474-z.

## Background

Alzheimer’s disease (AD) is an aging-associated neurodegenerative disorder, and as the most common form of dementia, afflicts approximately 5.8 million people in the United States [1]. It is estimated that 50 million people worldwide live with Alzheimer’s and other types of dementias [2]. As human populations age progressively, the economic burden AD poses to the healthcare system currently stands at $305 billion in the U.S., and in the near future, will grow immensely [1]. Typical onset of AD occurs after the age of 65 (late onset AD, LOAD), though less than 5 % of AD cases occur early (early onset AD, EOAD), while 1–2 % are inherited within families (familial AD) [3]. The primary clinical manifestations of the disease include profound cognitive decline, progressive memory loss, retrograde and anterograde amnesia, accompanied by severe histopathological changes, such as degeneration of the hippocampus and subsequent loss of cortical matter [4]. AD often manifests with additional comorbidities, such as movement and psychological disorders, as well as various sleep disturbances [1]. These heterogeneous symptoms confound diagnosis of AD in some cases [5]. Extensive molecular studies have revealed the pathological hallmarks of this malady: amyloid plaques comprised of amyloid-β (Aβ) peptides, and neurofibrillary tangles (NFT) containing hyperphosphorylated tau, which are used to categorize the disease stage (e.g. Braak stages) [6, 7]. Despite active investigation and drug development over decades [3, 8], and recent controversial approval of aducanumab (also known as Aduhelm) for AD treatment [9], precise causes of this brain degeneration are not fully understood, and a cure for this devastating disease still remains elusive.

The fundamental insights into AD pathogenesis come from complementary genetic/genomic and biochemical/proteomic studies. In 1984, Glenner and Wong isolated Aβ peptide from plaques in AD patients [10], which was later partially sequenced [11, 12], leading to subsequent gene cloning of β-amyloid precursor protein (APP) [13, 14]. This biochemical finding was later corroborated by mapping genetic mutations in causative AD genes, including *APP* in 1991 [15], and presenilins (*PSEN1/PSEN2*) in 1995 [16, 17]. The integration of biochemical and genetic evidence substantiates an intuitive molecular mechanism to AD, whereby the sequential proteolytic cleavage of APP by β-secretase (BACE1) and γ-secretase (containing PSEN1/PSEN2) produces amyloidogenic Aβ peptides [18–20]. Together, these results have ushered in the amyloid hypothesis, contending that Aβ species from APP cleavage play a central role in driving AD pathogenesis [21–23]. In parallel, hyperphosphorylated tau was purified as the dominant component of neurofibrillary tangles in AD brain tissues around 1986 [24–27]. Mutations to the tau gene (MAPT) were linked to several other neurodegenerative diseases, such as frontotemporal dementia (FTD) [28–30]. Again, biochemical and genetic evidence has established the tau hypothesis to propose its critical role in AD progression [31]. Experiments from the 2000s suggest that Aβ aggregates prior to cognitive defects, and later, downstream tau accumulation drives neurotoxicity [32–34]. The current paradigm has shifted from Aβ deposition towards understanding the toxicity of different Aβ forms, especially soluble Aβ oligomers [35]. Misfolded Aβ and tau may also transmit as “pathological seeds” during neurodegeneration [36].

Alternatively, a myriad of other models have been postulated: cholinergic [37, 38], calcium [39, 40], mitochondrial [41, 42], membrane trafficking [43, 44], inflammatory [45–47], lymphatic [48, 49], microorganism infection [50–52], neurovascular [53, 54], and cellular phase [55] hypotheses, although these alternative concepts are still entangled with the framework of amyloid and tau theories. The fact remains that Aβ may be essential, but not sufficient, to cause AD [56]. Nevertheless, the consensus molecular pathways, cellular circuits, and pathophysiological mechanisms mediating Aβ and tau toxicity are not fully understood yet.

Discoveries in the AD field have been accelerated by the innovations of large-scale sequencing technologies in genomics [57] and proteomics [58]. In addition to *APP*, *PSEN1*, and *PSEN2*, early genetic studies discovered the major AD risk gene of *APOE4* in 1993 [59, 60]. Current high-throughput genetic/genomic analysis revealed new risk genes, such as *TREM2* [61, 62] and *UNC5C* [63], and more than 160 possible risk loci linked to amyloid, tau, endocytosis, and immunity (collected in Supplementary Table S1) [64–68]. In 2014, the National Institutes on Aging initiated the Accelerating Medicines Partnership (AMP)-AD program to leverage multidisciplinary strategies across academia and industry, aiming towards the discovery of novel therapeutic targets and biomarkers [69]. The multi-omics approach offers an indispensable, systematic tool for understanding AD’s complexity. While the AD genetics field has been reviewed extensively over the years [70–73], here we intend to review the field of AD proteomics. Searching PubMed’s literature repository with terms “Alzheimer’s or Alzheimer” and “Proteomics or Proteome” returned more than 2,000 publications, and over 300 papers in 2020, although the AD proteomics field emerged at the turn of the millennia and has steadily increased in prominence. Much of the recent work endeavors to profile deep brain proteomes [74–77], analyze large sample sizes [78], dissect sub-proteomes [79–81], investigate complex PTM patterns [74, 82], and identify new biomarker candidates in biofluids [74, 75, 83–85]. We discuss the historical, current, and future status of protein analytical technologies and application, and provide a holistic view of the AD proteomic landscape by meta-analysis, unraveling new insights into AD pathogenesis and potential biomarkers.

## The Evolving Technologies of Protein Analysis and Mass Spectrometry

**Fig. 1:**
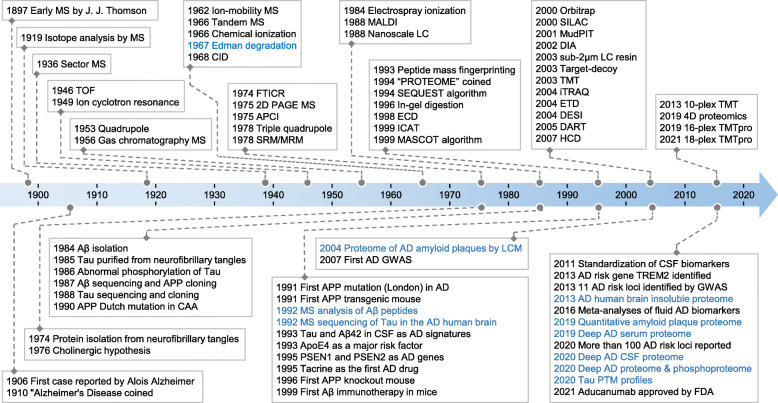
Major historical events in mass spectrometry and AD research. Edman degradation is also included. The AD proteomics studies are highlighted. The information is compiled from several online resources (https://www.nature.com/collections/aajfehieag, https://www.hupo.org/Proteomics-Timeline, https://masspec.scripps.edu/learn/ms, https://www.alzforum.org/timeline, and https://www.alzheimers.net/history-of-alzheimers) and references [74, 75, 79, 80, 82, 96–102]. MS detection instruments include sector MS, time-of-flight (TOF), quadrupole, Fourier-transform ion cyclotron resonance (FTICR), triple quadrupole, and orbitrap. Common ionization methods include atmospheric pressure chemical ionization (APCI), electrospray ionization (ESI), matrix-assisted laser desorption/ionization (MALDI), desorption electrospray ionization (DESI), and direct analysis in real time (DART). Biomolecules may be fractionated by nanoscale liquid chromatography (LC) with sub-2 μm resin to improve resolution, or by multi-dimensional protein identification technology (MudPIT). MS precursor ions can be fragmented by collision-induced dissociation (CID), electron-capture dissociation (ECD), electron-transfer dissociation (ETD), or higher-energy collisional dissociation (HCD). Quantitative strategies include two dimensional polyacryl amide gel electrophoresis (2D PAGE), isotope-coded affinity tag (ICAT), stable isotope labeling by/with amino acids in cell culture (SILAC), tandem mass tag (TMT), isobaric tags for relative and absolute quantitation (iTRAQ), and the data-independent acquisition (DIA) methods. Selected/multiple reaction monitoring (SRM/MRM) is a MS technique for analyzing pre-defined molecules. Database search tools contain SEQUEST, MASCOT, and the target-decoy strategy. CAA: cerebral amyloid angiopathy; LCM: laser capture microdissection; GWAS: genome-wide association study; CSF: cerebrospinal fluid; and FDA: the United States Food and Drug Administration

The major events contributing to mass spectrometry (MS)-based protein analysis during the last century are outlined (Fig. 1). Preceding MS-based proteomic analysis, Pehr Edman reported a technique (Edman degradation) for sequencing the amino acids of proteins in 1967 [86], but only up to 30 amino acids could be sequenced and each residue took approximately one hour [87]. It was this technique with which Wong et al. sequenced the first 28 residues in the Aβ peptides [11]. Several groups provided proof-of-principle evidence for sequencing amino acids of oligopeptides with commercial mass spectrometers that became available in the 1960s [88–91]. However, the MS application in protein sequencing was limited until the complete development of two Nobel Prize-winning methods for soft ionization in late 1980s [92]: matrix-assisted laser desorption/ionization (MALDI) [93] and electrospray ionization (ESI) [94]. MALDI was a popular ionization technique early on, and ESI is now the dominant method for proteomic analysis within complex mixtures [95]. The development of these technologies has made possible the high throughput analysis of proteins by mass spectrometry.

**Fig. 2:**
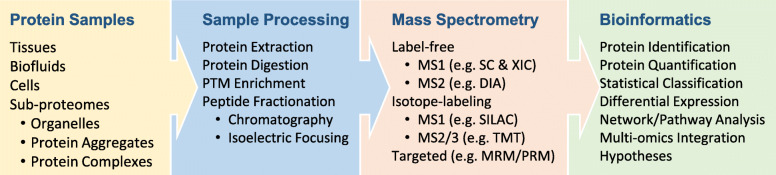
Protein samples are analyzed by pre-MS sample processing, MS data acquisition and post-MS bioinformatic data processing. Protein quantification can be achieved by label-free methods, such as spectral counting (SC), extracted ion current (XIC), and data-independent acquisition (DIA), or by isotope-labeling methods, such as SILAC and TMT. In addition, MS may also be operated to analyze targeted proteins/peptides by multiple reaction monitoring (MRM) or parallel reaction monitoring (PRM)

MS-based proteomic analysis often consists of three major steps (Fig. 2): (i) pre-MS sample processing, (ii) MS data acquisition, and (iii) post-MS bioinformatics; each step offers a wide variety of strategies to achieve final analytical goals of protein identification and quantification. For instance, one may choose top-down or bottom-up strategies, which analyze full-length proteins [103] or peptides (e.g., trypsin digested proteins) [104], respectively. Due to the highly diverse biochemical properties within the proteome, the top-down approach of analyzing all full-length proteins under one uniform condition is challenging. One of the most comprehensive top-down studies identified more than 3,000 protein isoforms from about 1,000 human genes [105]. In contrast, bottom-up proteomics relies on the analysis of peptides digested from full-length proteins, making the samples biochemically homogenous and thus improving proteome coverage to more than 10,000 proteins, although there is a gap to map identified peptides to proteins [106–108]. In bottom-up proteomics, digested peptides are usually separated by liquid chromatography (LC), ionized by ESI, and analyzed by tandem mass spectrometry (MS/MS or MS2) [109–111]. Isoelectric focusing is also used for high-resolution peptide separation [112]. It should be noted that MS2 itself is a powerful separation tool, enabling the selection of a single peptide from other co-eluted peptides based on the mass-to-charge ratio (*m/z*). Because of the sensitivity, throughput, robustness, and proteome coverage, the bottom-up method is commonly applied to untargeted proteome analysis.

Numerous bottom-up proteomics strategies are available, categorized into label-free and stable isotope labeling methods. The label-free methods have evolved from semi-quantitative spectral counting, which estimates the level of a protein by the total number of assigned MS2 spectra [113], to more accurate extracted ion currents in MS1 [114] or MS2 scans [115, 116]. The stable isotope labeling methods have begun with chemical protein labeling, such as Cys-reactive ICAT [117], amine-reactive dimethyl labeling [118], and then metabolic protein labeling like SILAC [119, 120], in which peptide/protein quantification is attained by MS1 peaks from the pooled samples with differential labels. However, the pooling increases the complexity of MS1 spectra, reducing the efficiency of peptide identification. To overcome this limitation, isobaric peptide labeling methods, such as iTRAQ [121], TMT [122] and DiLeu [123], have been developed to generate isobaric MS1 peaks and enable multiplexed tagging (e.g. 11-plex, 16-plex, 18-plex, and 27-plex TMT) [76, 124, 125]. After fragmentation of the isobaric peaks, different reporter ions are released for relative quantification in MS2/3 scans [126].

During MS data acquisition, the instrument is operated in the mode of data-dependent acquisition (DDA) [104], or an alternative mode of data-independent acquisition (DIA) [116]. In DDA, the spectrometer utilizes MS1 survey scans to select the most abundant peptide ions (e.g. top 10) in a small isolation window (e.g. 0.5-2 *m/z*), and then sequentially fragment them to generate MS2 scans. Though effective, some weak peptide ions are skipped in DDA, resulting in missing values. To alleviate this issue of undersampling, DIA sets up continuous isolation windows (e.g. 10–50 *m/z*) across the entire mass range and fragments all ions indiscriminately. The scanning of all DIA windows, however, increases time dependency. Therefore DIA was inefficient until rapidly scanning instruments became available [116]. Regarding MS instruments, early analyses utilized time-of-flight (TOF) mass spectrometers that were readily coupled with MALDI [92]. Later, triple quadrupoles (QqQ) and linear ion traps were popularized as low-resolution MS due to their cost effectiveness, while expensive Fourier-transform instruments (FTICR) were used for high-resolution detection [92]. Nowadays, with ion separation capacity provided by ion mobility spectrometry (IMS), TOF instruments have been significantly improved [127]. Moreover, the invention of compact Orbitrap instruments has revolutionized MS-based proteomics by offering a robust and high-resolution approach to proteome profiling [128].

Post-MS bioinformatics tools extract accurate information from raw MS data to protein identification and quantification. Large protein databases or MS spectral libraries may be searched with computational programs to assign MS2 spectra to peptides. A large number of search algorithms have been developed and reviewed elsewhere [129, 130]. One caveat to large-scale protein database search is the risk of false discovery, which has been addressed by the introduction of the target-decoy strategy to reduce the false discovery rate (FDR) of identified proteins (e.g. <1 %) [110, 131]. Following protein identification, quantification is often performed to assess protein abundances within samples, followed by differential expression (DE) and network analysis to derive testable hypotheses.

**Fig. 3:**
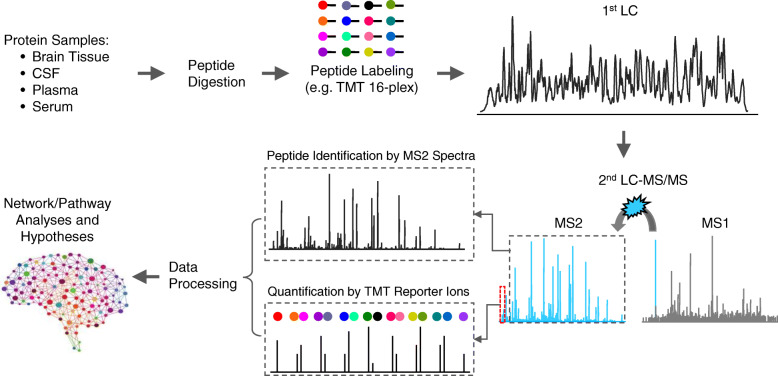
A deep proteomic workflow of TMT-LC/LC-MS/MS. The 16-plex TMT reagents are represented by different colors, which react with amine groups at peptide N-termini and lysine residues. In addition to the amine-reactive group, the isobaric TMT reagents also contain a reporter ion group and a balance group. The mass difference in the reporter group is offset by the balance group, enabling isobaric labeling and pooling. The pooled peptides are fractionated by LC/LC, and identified as mixed, isobaric precursor ions. After fragmentation, the TMT tags are cleaved between the reporter and balance groups, generating reporter ions for quantification

Overall, robust bottom-up proteomic methods require the seamless combination of pre-MS, MS and post-MS settings. The number of identified proteins is an indispensable metric for assessing proteomic techniques because crucial regulatory proteins are often present at low abundance in cells, which cannot be detected by a shallow proteomic analysis. To achieve ultra-deep proteome coverage [119], a common proteomics platform (e.g. TMT-LC/LC-MS/MS) utilizes multiplexed TMT labeling, two-dimensional HPLC (e.g. basic pH and acidic pH reverse phase liquid chromatography), and DDA in high resolution MS (Fig. 3) [117, 118]. This platform recently generated numerous deep AD proteomic datasets. Alternatively, LC-IMS-DIA-MS is a promising label-free platform, which combines ion mobility spectrometry, one-dimensional LC, and data-independent acquisition in MS to analyze about 10,000 proteins from brain tissue [132]. In addition to MS, proteins can be analyzed using specific affinity reagents such as antibodies (e.g. protein chips, ELISA, and proximity extension assay) [133–135] and aptamers (e.g. SOMAscan) [136]. These non-MS techniques are advantageous in processing a large number of samples, but current drawbacks of reagent availability and specificity prevent whole proteome coverage.

## Towards Unbiased Analysis of the AD Proteome

Evolving proteomic technologies have explored clinical AD specimens to elucidate the underlying biomolecular mechanisms of the disease in different time periods (Fig. 1). In the pre-genomic era, incomplete protein databases limited MS analysis to known AD proteins (e.g. Aβ and tau). LC-ESI-MS and MALDI-TOF-MS investigated the composition of amyloid plaques, identifying different Aβ peptides in soluble and insoluble forms [96, 137]. MS also identified abnormally modified tau species, including phosphorylation, acetylation, and deamidation [97]. With the completion of the Human Genome Project in 2003, the first comprehensive human protein database became available, providing the foundation for large-scale and unbiased proteomic analyses; however, it took another decade for proteomics technology to develop for matching the depth of the human proteome.

Given the early challenges to analyzing the whole proteome, sub-proteome analysis appeared to be an effective strategy. In 2004, Liao et al. characterized the amyloid plaque proteome from postmortem AD brain tissues through laser capture microdissection (LCM), a technique for dissecting and isolating a tissue region of interest [98]. Amyloid plaques labeled with thioflavin-S were captured, followed by protein extraction and MS analysis [138], identifying a total of 488 proteins. Comparison between the plaques and non-plaque tissues suggested 26 DE proteins enriched in the plaque area. This pilot study demonstrated that a large number of proteins may accumulate in amyloid plaques [139]. For simplicity, we use the term “amyloidome” to represent all biomolecules in amyloid plaques. Using a similar approach, Drummond et al. quantified over 900 proteins in the amyloidome from AD subtypes, reporting protein difference of rapidly progressive AD and sporadic AD [140]. In 2019, Xiong et al. re-analyzed the AD amyloidome in greater depth with LCM and the current TMT-LC/LC-MS/MS method, increasing the proteome coverage to more than 4,000 proteins, including 40 DE proteins highly enriched in the plaque region, including APOE and complement proteins [80].

Biochemical differential extraction of AD specimens offers an alternative means of enriching the aggregate proteome due to its low solubility [141, 142]. In 2009, Gozal et al. set out to analyze the detergent-insoluble proteome in AD; sequential fractionation in conjunction with gel electrophoresis and LC-MS/MS profiled 512 proteins, of which 11 proteins were increased in the AD samples [143]. Significant progress was made in 2013 wherein Bai et al., reported the most comprehensive analysis of the brain insoluble proteome, covering 4,216 proteins with 36 DE proteins in AD [79]. As expected, the most enriched proteins were Aβ, tau, APOE and complement components, together with proteins involved with RNA splicing, phosphorylation regulation, synaptic plasticity, and mitochondrial function. Surprisingly, the entire U1 snRNP splicing complex (e.g. U1-70 K, U1A, U1C, U1 snRNA and a U1-70 K cleaved fragment) is present in the insoluble proteome and form a new type of cytoplasmic tangle-like fibril in sporadic and familial AD cases [79, 144–147]. Together with concomitant RNA splicing defects revealed by transcriptomics [79, 148, 149], these results suggest the U1 snRNP pathology and its associated RNA splicing dysfunction in AD. Further studies in cell culture, fly and mouse models link splicing defects with the pathogenesis of Alzheimer’s disease [150–152].

In AD, synaptic loss occurs early and is highly correlated with cognitive impairment [153]; therefore, the synapse subproteome has been frequently visited [154–156]. The postsynaptic density (PSD) is an integral structure for synaptic function, organized by supramolecular complexes consisting of neurotransmitter receptors, scaffold proteins, and other regulatory constitutes [157]. Zhou et al. used ultracentrifugation and differential extraction to isolate PSD from AD brain, and analyzed 494 PSD components by the label free method [154]. More recently, two groups utilized the advanced TMT method to quantitate ~ 5,000 proteins in synaptic subproteome [155, 156]. In particular, Carlyle et al. examined 100 brains in different disease stages of AD patients, revealing that cognitive impairment is associated with significant metabolic changes and the increased inflammatory response [156].

Beyond subproteome studies, numerous studies attempted to characterize the whole proteomic changes in AD brain, using both 2D gels [158] or bottom-up MS to procure < 1,000 and 1,408-6,533 proteins [78, 159–162], respectively. For example, Johnson et al. profiled the proteome in more than 2,000 brains, and network analysis revealed a large number of AD-associated protein modules involved with synapse/neuron, mitochondrial function, sugar metabolism, extracellular matrix, cytoskeleton, and RNA binding/splicing [78]. These studies also identified protein modules enriched in neurons, microglia, and astrocytes [78, 160, 161]. In 2015, an advanced LC/LC-MS/MS approach enabled the identification of > 10,000 proteins in the AD brain [163]. Coupling this approach to a TMT labeling approach, deep proteome profiling became feasible [164, 165]. In 2020, Bai et al., presented such a study to compare 14,513 proteins in five groups of postmortem brain tissues: (i) controls with low plaques and tangles, (ii) controls with high pathology but no cognitive impairment, (iii) mild cognitive impairment (MCI), (iv) AD with high pathology, and (v) progressive supranuclear palsy (PSP) with only tau pathology [74]. This study identified 173 DE proteins, in which the vast majority were specific to AD when compared to the PSP cases. The DE proteins were clustered into three major co-expression patterns as derived from the weighted gene correlation network analysis (WGCNA) algorithm [166]: (i) an Aβ-correlated pattern (continuous accumulation from the control, MCI to AD), (ii) a tau-correlated pattern (steady from the control to MCI, but increased in AD), and (iii) a reversely correlated pattern with tau (stable from the control to MCI, but decreased in AD). Combining interactome and pathway analysis of the DE proteins revealed 17 altered pathways, including Aβ, WNT, TGF-β/BMP, G protein, integrin signaling, innate immunity, adaptive immunity, complement, cytoskeleton and extracellular matrix, iron homeostasis, membrane transport, lipid metabolism, protein folding and degradation, synaptic, neurotrophic and mitochondrial functions. The deep AD proteomics result indicates a broad, dynamic proteomic perturbation during AD progression [74].

**Fig. 4:**
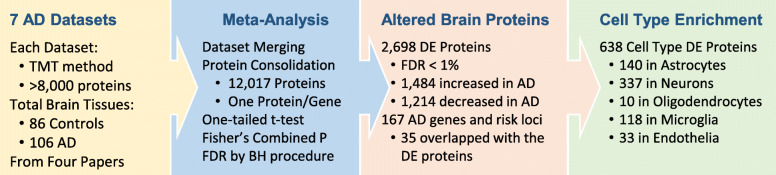
A meta-analysis integrating 7 deep AD datasets and identifying 12,017 proteins. In each dataset, the p value for control-AD comparison is derived by one-tailed t-test. The p values are combined by the Fisher’s method, followed by multiple-hypothesis correction using the Benjamini-Hochberg FDR procedure. A total of 2,698 DE proteins are accepted with the FDR cutoff of 1 %. Compared to 167 reported AD genes and risk loci, 35 are overlapped with the protein DE list. Based on cell type specific genes, the DE list contains 638 genes/proteins specific to all five major cell types in the brain

To date, three independent groups used the TMT-LC/LC-MS/MS platform to generate seven deep proteomic datasets (each identifying > 8,000 proteins) from a total of 192 AD and control cortical specimens (Fig. 4) [74–77]. These datasets offer an excellent opportunity for a meta-analysis to enhance the statistical power, compiled into a list of 12,017 unique gene products (one protein per gene, Supplementary Table S2). The log_2_(AD/control) ratio and associated one-tailed p-value were derived, followed by p-value combination using Fisher’s method and Benjamini-Hochberg FDR correction, resulting in 2,698 DE proteins (FDR < 1 %). Based on cell type specific gene profiles [167], the DE list contains 638 cell type specific genes/proteins, including 140 in astrocytes, 337 in neurons, 10 in oligodendrocytes, 118 in microglia, and 33 in endothelia (Supplementary Table S3), supporting the contribution of diverse cell types to AD pathogenesis.

## Functional Insights from the AD Proteomic Landscape

In addition to recapitulating the known accumulation of Aβ, tau and APOE in AD brain, the proteomic landscape reveals 1,484 upregulated proteins and 1,214 downregulated proteins (Fig. 4, Supplementary Table S2) [74–77]. Interestingly, the DE list contains 35 reported AD genes and risk loci, including APP, MAPT, CLU, APOE, ICA1, PTK2B, CD2AP, SNX32, ADAM17, FERMT2, CARHSP1, ANK3, ABI3, PLEKHA1, BCKDK, GRN, COX7C, TMEM163, CNTNAP2, ADAMTS1, NDUFAF7, SEL1L, RTFDC1, AGRN, ICA1L, SPRED2, HLA-DRB1, INPP5D, TPBG, PLCG2, IDUA, CTSH, PRKCH, PFDN1, and SHARPIN (Supplementary Table S1, ranked by protein false discovery rate). Along this line, a proteome-wide association study (PWAS) was reported to integrated GWAS data with brain proteomics results to determine 11 causal AD genes [168].

Proteome-transcriptome comparison suggests both RNA-dependent and RNA-independent expression changes in AD [74, 75]. Strikingly, these RNA-independent DE proteins are often highly correlated with the Aβ level, and are enriched in amyloidome, such as MDK, PTN, NTN1, SMOC1, SFRP1, SLIT2, HTRA1, and FLT1 [74, 80, 169]. Their RNA independence was validated through complementary proteomic and transcriptomic analysis of 5xFAD mice, an AD mouse model of amyloidosis. MDK, NTN1, and SFRP1 were also shown to bind directly to Aβ peptide [74] [169]. Given that amyloid plaques grow up to 100 μm in size, the enormous three-dimensional complexity, diverse cellular structures, and vasculature [170] of these plaques may create a quasi-cellular “black hole”, trapping within itself a broad cohort of associated proteins which profoundly impacts protein turnover. Among these proteins in amyloidome, SFRP1 (a regulator of WNT signaling) was reported to affect the formation of Aβ oligomers, as SFRP1 inhibition reduces plaque formation and partially rescues cognitive deficits in an AD mouse model (APP/PS1 mice), supporting its role in AD pathogenesis [169].

**Fig. 5:**
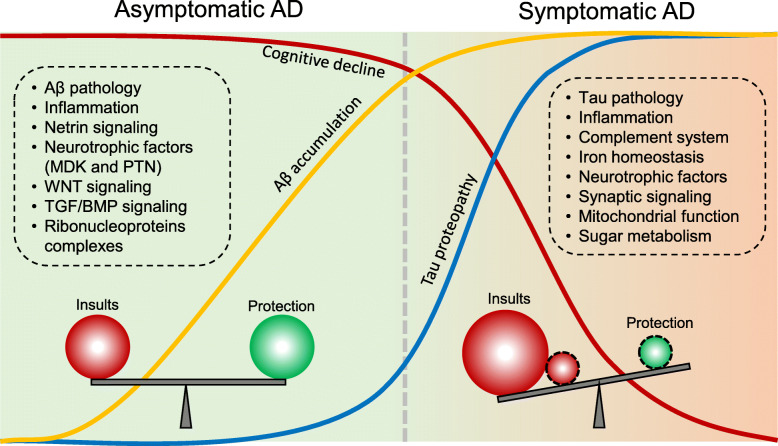
The equilibrium model of deleterious and protective factors during AD disease progression. Among the biological processes and cellular pathways activated at the asymptomatic stage of AD, some may exert protective roles. However, with exacerbation of the harmful insults during disease progression, the protective effect is exhausted. The resulting imbalance leads to neuronal degeneration and the onset of clinical symptoms. The pathway information is extracted from current AD proteomic studies [74, 75, 78, 161]

AD development spans a long prodromal stage before progressive neurodegeneration, implicating a resilient mechanism to Aβ toxicity in human brain, followed by exacerbated insults to outweigh the resilience and drive irreversible degeneration [55], illustrated in an equilibrium model (Fig. 5). Prior to the onset of AD, some of the identified DE proteins might play protective roles, such as netrin-1 (NTN1), netrin-3 (NTN3), midkine (MDK), pleiotrophin (PTN), hepatocyte growth factor (HGF), and WNT5B, particularly in the human resilient cases that display high Aβ pathology but without clinical symptoms [74]. Literary evidence corroborates this evaluation, such as in the case of intracerebroventricular administration of netrin-1 which improved working memory in AD mice [171]. The netrin receptor UNC5C was identified as an AD risk gene [63], and NTN5 (another netrin family member) was located in an AD risk loci in a GWAS [68]. Midkine and pleiotrophin, in the same family of neurite growth-promoting factor, were shown to directly interact with Aβ with high affinity, possibly interfering with Aβ oligomerization and diminishing its toxicity [172, 173]. Upregulation of HGF signaling may enhance synaptogenesis, thus recompensing synaptic loss in AD [174]. Furthermore, the WNT pathway was proposed to promote the viability of microglia, which was induced by the AD risk gene TREM2 [175].

The transition from MCI to AD may be induced by the upregulation of detrimental events and the diminishing of protective events, consubstantial with marked increase of tau pathology. The DE list (Supplementary Table S2) contains a large number of complement proteins (C1QA, C1QB, C1QC, C1QL1, C1QTNF5, C1R, C1S, C3, C4A, and C4B) [74, 75]. Complement components are immensely elevated in the aggregated subproteome of the AD brain [73]. The complement system activates microglia to trim synapses in AD [176].The complement genes CR1 and C7 were identified as AD risk genes in large-scale GWAS analyses [64, 177]. Deleting C3 in the AD (APP/PS1) mice was shown to rescue synapse loss and memory decline [178]. Knocking out of C3aR (C3a receptor) in a tauopathy model (PS19 mice) also reduced inflammation, synaptic deficits and tau pathology [179]. Therefore, the complement system influences multiple molecular and cellular events during the progression of Alzheimer’s disease [180].

In contrast, many neurotrophic factors are decreased during the transition from MCI to AD, such as VGF, BDNF, NRN1, and CRH [74, 75]. Notably, the former four neurotrophic factors are connected together surrounding the BDNF hub in protein-protein interaction network. BDNF has been long viewed as a component related to cognitive deficit, aging and AD [181]. Multiscale causal network analysis of AD multi-omics datasets using the RIMBANET software ranks VGF as a master regulator [182]. The overexpression of VGF in the 5xFAD mice attenuates the pathology and improves memory performance. NRN1 can enhance neurite outgrowth and synapse maturation, and administration of recombinant NRN1 protein in a mouse model (Tg2576) improves synaptic plasticity [183]. In addition, NPTX2 (a binding protein of AMPA type glutamate receptors) is markedly decreased in AD. Its gene knockout in the AD (APP/PS1) mice leads to reduced GluA4 expression and increased neuron excitability [184]. Collectively, the decrease of these key proteins may be a pathophysiological mechanism contributing to synaptic failure and cognitive impairment in AD.

Although we use a simple equilibrium model to discuss the roles of identified DE proteins during AD progression (Fig. 5), it is highly possible that the function of these AD-associated proteins is multifactorial during the long-lasting course of disease development, and whether they play a protective or detrimental role might be dependent on the temporal, regional and cellular contexts.

## Post-translationally Modified Proteomes in AD

Protein functionality *in vivo* is tightly regulated by a myriad of posttranslational modifications and dynamic protein-protein interactions. In neurodegenerative diseases exhibiting tauopathy, structurally distinct conformers of tau fibrils are characterized to classify disease subtypes [185]. Tau aggregation is associated with extensive PTMs, including phosphorylation, ubiquitination, acetylation, methylation, glycosylation, sumoylation, oxidation and cleavage [186–188]. A plausible explanation to these disease specific tau conformers could be hidden in the realms of PTMs. Arakhamia et al. utilized MS and cryo-electron microscopy (cryo-EM) to map PTMs on the structures of tau filaments, and found that ubiquitination of tau may contribute to fibril diversity [189]. Recently, tau was extracted from 32 AD patients and characterized by MS and seeding activity for protein aggregation. The seeding capacity was found to significantly correlate with phosphorylation at T231, S235 and S262 sites [190].

Tau acetylation is known to elevate during early and moderate Braak stages of tauopathy and may slow down tau degradation [191]. Acetylation of K280/K281 sites was suggested to promote the aggregation of tau [192]. The status of tau acetylation is regulated by the p300 acetyltransferase and the SIRT1 deacetylase. Chemical inhibition of the p300 activity reduced the acetylation level of tau, and thus eliminated tau-related pathology, suggesting a possible treatment strategy to alleviate the tauopathy [191].

More recently, Wesseling et al. reported the most comprehensive tau PTM analysis in AD brains by an array of MS strategies. A total of 95 modification events (55 in phosphorylation, 17 in ubiquitination, 19 of acetylation and 4 in methylation) were identified on multiple tau isoforms from 42 control and 49 AD cases. Using tau internal standards, the stoichiometry of some PTM sites was also measured. The PTM events may occur in order, and contribute to tau aggregation and seeding activity by altering surface charge. These profiles of tau PTMs in AD patients reveal molecular heterogeneity and disease stages.

Globally, Bai et al. identified 46,612 phosphopeptides (34,173 phosphosites in 7,083 proteins) in brain tissues at different stages of AD subjects by the combination of phosphopeptide enrichment and TMT-LC/LC-MS/MS approach, revealing 873 DE phosphopeptides in 398 proteins [74]. The study also identified in AD hyperphosphorylated tau (56 phosphosites) and osteopontin (SPP1), a glycoprotein in the immune response. Interestingly, the IKAP algorithm [193] was used to derive the activities of 186 kinases from the phosphoproteome, suggesting 28 differential kinase activities, covering all known 11 tau kinases [188]. Integration of kinase activities and levels collectively indicate the activation of MAP kinase signaling in AD [74]. Using the similar method, Ping et al. reported another independent quantitative dataset of AD phosphoproteome (33,652 phosphosites in 8,415 proteins) [194]. The phosphoproteome profiling provides another layer of proteomic information during AD development.

In addition, the global analysis of protein ubiquitination in AD was reported, covering 4,291 ubiquitinated sites in 1,682 proteins, in which more than 800 sites were altered in AD [195]. Specific polyubiquitination chains (Lys11, Lys48 and Lys63) were also found to accumulate in AD brain tissues [196]. These data implicate the deregulation of the ubiquitin system in AD. Protein N-glycosylation is one of the most prevalent PTMs in cells [197]. Zhang et al. profiled 2,294 N-glycosylation sites in 1,132 proteins in human brains to show AD-associated changes of 178 N-glycosylation sites, suggesting the aberration of protein N-glycosylation in the disease [198].

In summary, current MS platforms and enrichment strategies enable either focused or proteome-wide analysis of PTMs in AD specimens. Large datasets have been emerging, especially in phosphoproteome. A plethora of PTM information is also available online (www.phosphosite.org), although the data are not AD-specific. Together, these comprehensive PTM datasets will be valuable for investigation of biochemical signaling pathways during AD pathogenesis.

## Proteomics-Based Biomarker Discovery in AD

Exciting progress has been made toward MS-based proteomic profiling of cerebrospinal fluid (CSF) for biomarker discovery [74, 75, 83–85, 199]. In many proteomic experiments, highly abundant proteins tend to mask proteins present in low amounts, so immunodepletion of these abundant proteins is a frequently used procedure for improving the detection of proteins of low abundance. Sathe et al. reported a pilot deep CSF study from 5 control and 5 AD cases, in which CSF samples were first immunodepleted to remove 14 most abundant proteins, and then analyzed by the TMT-LC/LC-MS/MS approach. The study quantified 2,327 proteins with 139 DE proteins, including MAPT, NPTX2, VGF, GFAP, NCAM1, PKM and YWHAG [83]. Higginbotham et al. also used immunodepletion to enhance the depth of CSF proteome, profiled 2,875 proteins from 20 control and 20 AD cases, revealing 528 DE proteins, including MAPT, NEFL, GAP43, FABP3, CHI3L1, NRGN; VGF, GDI1 and SMOC1 [75].

Wang et al. however performed extensive LC fractionation to bypass immunodepletion, and profiled 5,941 CSF proteins from 5 control and 8 AD cases, significantly increasing the coverage of CSF proteome [74, 85]. The study yielded 355 DE proteins, revealing increases to SMOC1 and TGFB2, and decreases to a large group of mitochondrial proteins. Alternatively, Bader et al. developed an advanced label-free platform to profile undepleted CSF samples from three independent cohorts of similar size (totaling 109 control and 88 AD cases). The study analyzed 1,445, 1,478 and 1,483 proteins in the three cohorts, highlighting the three DE proteins of tau, SOD1, and PARK7, which are also linked to neurodegeneration by genetic data [84]. In all above studies, the conclusions were also supported, at least partially, by validation experiments using antibody-based detection, targeted MS approaches, or replicated proteomic analysis.

**Fig. 6:**
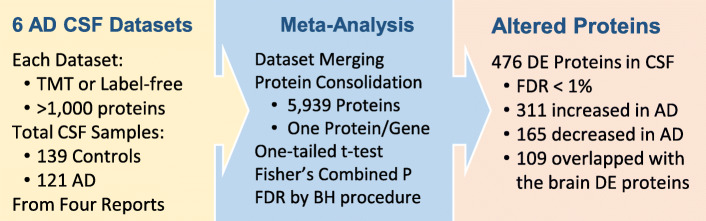
A meta-analysis integrating 6 deep CSF datasets and identifying 5,939 proteins. Using the same method in Fig. 4, individual and combined p values for control-AD comparison are computed, followed by the FDR analysis. With the FDR cutoff of 1 %, 476 DE proteins are accepted, 109 are overlapped with the list of DE proteins in AD brain tissues

The meta-analysis of these six deep CSF datasets above (each > 1,000 proteins, summed 260 AD and control subjects) [74, 75, 83–85] yielded a list of 5,939 proteins, which includes 311 upregulated and 165 downregulated proteins in AD (Fig. 6, Supplementary Table S4, FDR < 1 %). To enhance the selection of specific CSF biomarkers, integrating CSF and brain proteomes is a common strategy [74, 75, 78, 85]. We then overlapped these DE proteins in CSF with the brain proteome (Supplementary Table S2), yielding a core table of 65 upregulated proteins (e.g. MAPT, SMOC1, HTRA1, PDLIM5, PRDX6, RUVBL2, CALB2, ARFGAP3, SPP1 and DPCD) and 44 downregulated proteins in AD (e.g. NPTX2, VGF, PDHA1, NDUFV1, NDUFA2, NDUFA12, NDUFA13, NDUFS3, ATP5B and ATP5J, Supplementary Table S5). The meta-analysis (Supplementary Table S4 and S5) provides a resource of AD CSF proteome for future validation.

Moreover, Wang et al. compared human CSF and 5xFAD mouse CSF datasets, pointing to 11 shared DE proteins (e.g. SOD2, PRDX3, ALDH6A1, ETFB, HADHA, and CYB5R3) that may be induced by amyloidosis [85]. Higginbotham et al. summarized the CSF DE proteins into synaptic, vascular, myelin, immunological, and metabolic panels, and applied the five biomarker panels to the classification of asymptomatic AD subjects into two subgroups [75]. In agreement with the heterogeneity of AD-related cases, three molecular subtypes are implicated by transcriptomics [200] and CSF proteomics [134].

In contrast to CSF analysis, deep MS-based profiling of AD plasma/serum samples is sparse, because molecular changes in the brain may not be readily detected in the blood due to the blood–brain barrier. The unique blood composition also imposes an analytical challenge with an extremely large dynamic range, from albumin (~ 50 mg/ml) to interleukin-6 (~ 4.2 pg/ml). Dey et al. reported an ultra-deep analysis of undepleted human sera from 5 control and 6 AD cases by TMT and exhaustive LC/LC-MS/MS, analyzing 4,826 proteins but still missing the layer of proteins at the lowest abundance (e.g. Aβ peptide and tau) [99]. Nevertheless, overlapping of serum, CSF and cortex proteomes identified 37 DE proteins including 22 mitochondrial proteins, suggesting a consistent mitochondrial signature in AD [85].

Extracellular vesicles (EV) enable the transport of brain proteins to the blood, which emerge as an alternative class of specimens by MS analysis. EVs are highly heterogeneous, nano-sized lipid vesicles released to the extracellular environment for cell communication [201], secreted by almost any cell types including neurons, astrocytes, and microglia [202]. EVs carry and spread AD-related proteins including APP, Aβ peptides, and tau [203, 204]. Increased levels of Aβ_42_/Aβ_40_ ratio, APP, Aβ monomer and oligomer forms, tau and tau phosphorylation were reported in EVs isolated from the plasma, CSF, and brain cells of AD patients and mouse models [202, 205, 206]. With accumulating evidence suggesting the correlation of EV protein components with AD, we envision more comprehensive analysis of EV proteomics for AD biomarker discovery.

In addition to untargeted search of biomarker candidates by MS, the targeted trials of known pathological markers (e.g. Aβ and tau) in CSF and blood have been ongoing and successful. For example, the CSF tests of Aβ and phosphorylated tau have been accepted by the National Institute on Aging and the International Working Group for New Research Criteria for the diagnosis of AD and MCI [207, 208]. Recent CSF tests of pTau at T181, T217 or T231 residues showed high accuracy [209]. These analyses have been further extended to blood tests. The MS-based blood test of Aβ peptides (i.e. PrecivityAD™) is commercially available. Targeted MS methods were also developed to evaluate pTau levels at T217 and T181 residues in CSF and blood [210, 211]. The pTau data were consistent with PET imaging analysis of amyloid and tau, as well as clinical stage of AD. Although the pTau analysis is promising, its concentration in blood is extremely low (< 1 pg/ml) [211], requiring antibody-affinity enrichment, or highly sensitive antibody-based SIMOA kits [209]. Further independent and large-scale trials are required to evaluate the sensitivity and specificity of pTau biomarkers.

## Conclusions and Perspectives of AD Proteomics-based Systems Biology

In systems biology, biological and disease phenotypes are viewed as emergent properties regulated by components in both spatial and temporal dimensions, and their interactions confer functional consequences in a hierarchical, multi-scale system at structural, molecular, organellar, cellular, tissue, organ, and organismal levels. In this framework of systems biology, based on the introduction of recent major AD proteomics studies, we discuss the technical challenges and scientific questions in the field remaining to be resolved.

The sample quality in AD proteomics is unavoidably affected by many confounding factors, such as age, gender, postmortem interval (PMI), ischemia, etc. [212, 213]. The confounding effect on modified proteomes (e.g. phosphorylation and ubiquitination) is larger than that on the whole proteome, because protein modifications are highly transient and dynamic. While age- and gender-matched cases are selected for proteomic comparison, the effect of other confounding factors (e.g. PMI and ischemia) may be addressed by control experiments in animals [74, 214], and normalized by regression analyses [75]. Sample size is another critical parameter in AD proteomics, affected by biological and experimental variations [215]. Proteomic studies with a limited sample size often lead to biased conclusions that cannot be repeated in other studies. Reliable proteomic results should be consistent in multiple patient cohorts analyzed by different research groups.

In human brain, it is estimated that ~ 16,000 genes are expressed [163] to produce millions of proteoforms, largely attributed to RNA alternative splicing and PTMs [216]. For example, a large number of Aβ and tau proteoforms are present in AD brains due to the combination of protein modifications and proteolytic events. Although the bottom-up proteomics detects a large portion (more than 12,000 gene products, Supplementary Table S2) of brain proteome, mapping all intact proteoforms is not straightforward, as protease digestion in the bottom-up approach causes the loss of proteoform data. One may utilize the top-down MS to characterize proteins of interest, such as proteoforms of Aβ species [217] and tau proteins [218]. Innovative structural MS technologies have been developed to analyze protein structures of purified proteins, protein complexes, and even thousands of proteins [219–222], and will be applied to dissect structural changes in AD at a global scale. Eventually, the integration of bottom-up, top-down, and structural MS approaches will provide a more comprehensive view of the proteotype (defined as the state of a proteome linked to a specific phenotype) in AD patients [58]. In addition, continuously enhancing throughput, sensitivity, and affordability in proteomics is necessary. This will improve the depth and breadth of detected proteomes, and make it possible to handle large sample sizes to overcome limitations associated with protein dynamic range and clinical sample variations.

Cellular components in the brain are highly heterogeneous including different cell types in diverse cellular states of homeostasis or transition states. However, such cellular heterogeneity is masked by conventional population-based (or bulk) analyses. Recent development of single cell (SC) omics technologies (especially scRNA-seq) has allowed researchers to sample gene expression from the whole transcriptome at the single cell resolution extensively in AD animals and human brain tissues [223–228]. The resulting unbiased and holistic view of both molecular (e.g., gene expression) and cellular (i.e., single cell) dimensions enables identification of new cell (sub) populations, and those that associate with pathogenesis will be highlighted and further explored. With rapid development of single cell proteomics [229–231], single cell type proteomics [232, 233], and single-molecule protein sequencing technologies [234], the application to the AD field is expected in the near future.

It is critical to emphasize that there is a gap between generating proteomics data and discovering disease drivers, because proteome profiling only reveals disease-correlated components, but correlation does not imply causation. Significant investments are required to establish a cause-effect relationship in disease models and human patients. With recent identification of a large number of DE proteins in AD, the next step is to identify and validate the underlying mechanisms contributing to the molecular changes. Master regulators (e.g., transcription factors, kinases or other signaling proteins) that drive such changes are yet to be identified, partially because their expression is spatially and temporally restricted with their low abundances [235], and their functions are likely to be regulated by PTMs and/or protein-protein interactions which are largely missing. Hence it may be difficult to define some of these master regulators directly by gene/protein expression profiles. Alternatively, the driver activity can be inferred by summarizing the expression alteration of downstream target genes via systems biology approaches [236]. This network-activity based approach [237, 238], and other multi-omics network analyses [182, 239] will unveil disease drivers for AD pathogenesis. The novel disease drivers will be studied by interactome profiling and genetic approaches in cellular (e.g. induced pluripotent stem cells) [240] and animal models [241].

In summary, current deep proteomics studies have already profiled the brain and biofluids at an unprecedented scale, raising many novel hypotheses for subsequent validation. It is notable that AD is an irreversible neurodegeneration and a nearly end-stage disease, in which many cellular pathways and biological processes are perturbed. With further development in the MS-based or non-MS proteomics approaches, it is not surprising to see even more molecular alterations and characteristic proteins discovered. In parallel, it is anticipated that novel AD models are to be developed following the hypotheses from these molecular insights here we have discussed, providing potential therapeutic strategies and biomarkers for AD and subtypes of AD.

## Supplementary Information

**Additional file 1:**

## Data Availability

Not applicable.

## Abbreviations

AD: Alzheimer’s disease
CSF: Cerebrospinal fluid
EV: Extracellular vesicle
MS: Mass spectrometry
LC/LC-MS/MS: Two-dimensional liquid chromatography coupled with tandem mass spectrometry
TMT: Tandem mass tag
