# Correction to: Proteomic landscape of Alzheimer’s Disease: novel insights into pathogenesis and biomarker discovery

**DOI:** 10.1186/s13024-021-00493-w

**Published:** 2021-10-20

**Authors:** Bing Bai, David Vanderwall, Yuxin Li, Xusheng Wang, Suresh Poudel, Hong Wang, Kaushik Kumar Dey, Ping-Chung Chen, Ka Yang, Junmin Peng

**Affiliations:** 1 Departments of Structural Biology and Developmental Neurobiology, St. Jude Children’s Research Hospital, Memphis, TN 38105 USA; 2 Center for Proteomics and Metabolomics, St. Jude Children’s Research Hospital, Memphis, TN 38105 USA; 3 Current address: Center for Precision Medicine, Department of Laboratory Medicine, Nanjing Drum Tower Hospital, The Affiliated Hospital of Nanjing University Medical School, Nanjing, 210008 Jiangsu China; 4 Current address: Department of Biology, University of North Dakota, Grand Forks, ND 58202 USA

**Correction to: Mol Neurodegener 16, 55 (2021)**

**https://doi.org/10.1186/s13024-021-00474**-z

The original article [1] contained an error in Corresponding Authorship designation.

This has since been corrected.
